# Self-medication in Primary Dysmenorrhea among Undergraduate Students in a Medical College: A Descriptive Cross-sectional Study

**DOI:** 10.31729/jnma.7816

**Published:** 2022-12-31

**Authors:** Ruchi Shrestha, Mukta Singh Bhandari, Sony Shakya Shrestha, Jyoti Tara Manandhar Shrestha, Upama Shrestha

**Affiliations:** 1 Department of Pharmacology, Kathmandu University School of Medical Sciences, Dhulikhel, Kavre, Nepal; 2 Department of Community Medicine, Kathmandu University School of Medical Sciences, Dhulikhel, Kavre, Nepal

**Keywords:** *dysmenorrhea*, *menstruation*, *prevalence*, *self medication*

## Abstract

**Introduction:**

Primary dysmenorrhea is painful menstruation in women with normal pelvic anatomy, usually beginning during adolescence, primarily associated with a normal ovulatory cycle. There is an increased likelihood of self-medication among medical students. The aim of this study is to find out the prevalence of self-medication in primary dysmenorrhea among undergraduate students in a medical college.

**Methods:**

A descriptive cross-sectional study was conducted in a medical college among undergraduate female students with primary dysmenorrhea from 1 February 2022 to 31 May 2022. Ethical approval was obtained from the Institutional Review Committee of the same institute (Reference number: 254/2021). Convenience sampling was done. Data were collected through a selfadministered questionnaire. Point estimate and 95% Confidence Interval were calculated.

**Results:**

Among 213 students with primary dysmenorrhea, self-medication was found to be in 78 (36.62%) (30.15-43.09, 95% Confidence Interval)). Among all the self-medications used, mefenamic acid was most common, used by 45 (57.69%) students, followed by paracetamol 11 (14.10%).

**Conclusions:**

The prevalence of self-medication practice in primary dysmenorrhea among undergraduate students was lower when compared to similar studies done in similar settings.

## INTRODUCTION

Primary dysmenorrhea is a lower abdominal pain that occurs during the menstrual cycle, which is not associated with other diseases or pathology.^1^ Dysmenorrhea has a debilitating effect on the quality of life among affected female adolescents.^2^ In female students, the condition is associated with diminished quality of life, absenteeism and a drop in academic performance, with a significant socioeconomic impact.^3-5^

Self-medication practices are very common among medical students, facilitated by the easy availability of drugs, and information from textbooks and seniors.^6^ Commonly used drugs as self-medication include nonsteroidal anti-inflammatory drugs (NSAIDs), followed by drugs for gastritis, cough remedies and antimicrobials.^7^

The objective of this study was to find out the prevalence of self-medication in primary dysmenorrhea among undergraduate students of a medical college.

## METHODS

A descriptive cross-sectional study was conducted among undergraduate female students studying at Kathmandu University School of Medical Sciences (KUSMS) from 1 February 2022 to 31 May 2022. Ethical approval was obtained from the Institutional Review Committee of KUSMS (Reference number: IRC-KUSMS 254/2021). Before the commencement of the study, participants were explained the objective of the study and written consent was taken. Female students studying in MBBS, BDS, B.Sc Nursing, BNS and BPT with complaints of dysmenorrhea were included in the study. Married students, students with known pelvic pathology, and those who refused to participate were excluded from the study. Convenience sampling method was used. The sample size was calculated using the following formula:


$$\begin{array}{ll} \text{n} & ={\text{Z}}^{2}\times \frac{\text{p}\times \text{q}}{{\text{e}}^{\text{2}}}\\ & =1.96^{2}\times \frac{0.84\times 0.16}{0.05^{2}}\\ & =207 \end{array}$$

Where,

n = minimum required sample size
Z = 1.96 at 95% Confidence Interval (CI)
p = prevalence of self-medication in dysmenorrhea among students, 84.7%^8^
q = 1-p
e = margin of error, 5%

The calculated sample size was 207. However, 213 samples were taken.

Semi-structured questionnaire were developed after reviewing previously published related studies with few modifications.^2,9,10^ Questionnaire was divided into three parts. First part included demographic profile of students. Second part contained information regarding menstruation and features of dysmenorrhea which included age at menarche, regularity of menstrual cycle, presence or absence of dysmenorrhea, severity of pain, accompanying symptoms and features experienced during dysmenorrhea. Third part included questions related to self-medication practice and home remedies used to relieve dysmenorrhea along with information they possessed regarding self-medication they take during dysmenorrhea.

The Andersch and Milsom scale was used to determine the severity of dysmenorrhea according to series of grades.^11^ Grade 0 (menstruation is not painful and daily activity is unaffected), grade I (menstruation is painful but seldom inhibits normal activity, analgesics are seldom required and mild pain), grade II (daily activity affected, analgesics required and give relief so that absence from work or school is unusual and moderate pain) and grade III (activity clearly inhibited, poor effect of analgesics, vegetative symptoms and severe pain).

The selected respondents were explained about the purpose of the study. Questionnaire was submitted and collected during the lunch hour without disturbing any of their classes. Participants were requested to complete the questionnaire in the presence of the principal investigator. Each participant was allotted 15 minutes to answer the questionnaire which they felt appropriate to answer. They were informed about confidentiality of data that it would not be used for anything except for the study purpose.

Data were entered and analysed using IBM SPSS Statistics version 16.0. Point estimate and 95% CI were calculated.

## RESULTS

Among 213 students with primary dysmenorrhea, selfmedication in primary dysmenorrhoea was found in 78 (36.62%) (30.15-43.09, 95% CI). Only 8 (3.75%) sought for medical consultation. Highest prevalence of selfmedication was seen among grade II dysmenorrheic students 35 (44.87%) (Table 1).

**Table 1:** Severity of dysmenorrhea among selfmedication (n= 78).

	Grade I n (%)	Grade II n (%)	Grade III n (%)
Self-medication	25 (32.05)	35 (44.87)	18(23.08)

Among all self-medication used, mefenamic acid was most common, used by 45 (57.69%) students (Table 2).

**Table 2:** Most commonly used drugs for self-medication (n= 78).

Drugs used for self-medication	n (%)
Mefenamic acid	45 (57.69)
Paracetamol	11 (14.10)
Combination of Paracetamol and ibuprofen	9 (11.54)
paracetamol+mefenemic acid	8 (10.26)
Others^*^	5 (6.41)

* Others: diclofenac, nimesulide, drotaverine, ibuprofen

Among all the students who took self-medication 78 (36.62%), also used home remedies. Among the home remedies used by self-medicated students, drinking hot water was the most common (Table 3).

**Table 3:** Commonly used home remedies (n= 78).

Home remedies	n (%)
Drinking tea/coffee	35 (44.87)
Drinking hot water	73 (93.59)
Massage	25 (32.05)
Exercise	12 (15.38)
Hot water bag	68 (87.18)
Rest	61 (78.20)
Others^**^	3 (3.85)

** Others: sweets intake, using patuka

Mean age of the students with self-medication was 20.70±1.43 years. Mean age for menarche was 12.86±1.41 years, with the age range of 9-17 years. Positive family history of primary dysmenorrhea was present in 47 (60.26%) students. Regular menstruation was reported in 58 (74.36%) students. Average pain duration was 2.18±0.96 days. Most common accompanying symptoms and features were fatigue and emotional instability, which were reported in 59 (75.64%) and 50 (64.10%) students respectively (Table 4).

**Table 4:** Accompanying symptoms and features in dysmenorrheic students with self-medication (n= 78).

Accompanying symptoms	n (%)
Nausea	30 (38.46)
Vomiting	16 (20.51)
Dizziness	27 (34.61)
Headache	28 (35.90)
Fatigue	59 (75.64)
Bloated stomach	55 (70.51)
Eating disorders	33 (42.31)
Accompanying features	n (%)
Stress	29 (37.18)
Depression	6 (7.69)
Emotional instability	50 (64.10)
Limited daily activities	35 (44.87)
Decreased concentration	44 (56.41)
Decreased social activities	37 (47.43)
Absenteeism	6 (7.69)

## DISCUSSION

Self-medication practice was found in 78 (36.62%) out of 213 students. Self-medication practices are very common in medical students, facilitated by the easy availability of drugs, and information from textbooks and seniors.^6^ Other reasons may be mildness of disease, cost effectiveness, convenience, quick relief in common illness, shortage of time to consult a doctor, and confidence in self-diagnosis.^6,12^ Self-medication results in wastage of resources, and generally causes serious health hazards such as adverse drug reactions, prolonged suffering and drug dependence, increases resistance of pathogens in case of antibiotics.^6^

In our study, more than one third of the student reported self-medication for dysmenorrhea. Previous studies showed frequency of 41.7%, 61.7% and 70% for self-medication in dysmenorrheic students.^13-15^ A total of 23.07% self medicated sudent had grade III dysmenorrhea, 44.87% were suffering from grade II pain and 32.05% of those with grade I pain. In a study conducted in Spain, a total of 86.8% of students suffering from grade III took self-medication compared to 61% of students with grade II pain and 35.7% of those with grade I pain.^13^ In an another study from India, 32.9% of students with grade III pain were found to be self-medicating compared to 47.4% with grade II pain and 19.7% with grade I pain.^14^ In our study, only a small proportion of students reported to have sought medical advice, instead most of them were self medicating without doctor's consultation. This may be because young females may feel shy to go to doctor for medical consultation or they may believe that the painful periods are normal with no need of seeking medical advice.

In our study, among various self-medication used, mefenamic acid was used by more than half of the students followed by paracetamol. In a study conducted in India suggested that most commonly used drugs for self-medication in dysmenorrhea were dicyclomine in 35%, followed by mefenamic acid in 26%.^15^ In a study done in Serbian medical students, most commonly used group of drugs were ibuprofen (53.03%), and diclofenac (10.61%).^16^ In an another study in Nepal, commonly used drugs for self-medication for dysmenorrhea was reported to be mefenamic acid 48%, followed by ibuprofen 20.3% and paracetamol 16.3%.^17^ Previous study have showed that self-medication practice was higher among students from medical faculty as compared to students from non-medical faculty.^18^ However, an another study from Hongkong reported that more than half of the self-medicated women with dysmenorrhea were particularly nonmedical students.^19^

The present study showed that most of the students who were self-medicated for dysmenorrhea also used home remedies as a non-pharmacological treatment option. In our study, among the home remedies used in self-medicated students, more than two-thirds reported taking hot water and using hot water bag for managing dysmenorrhea. As the severity of pain increased, it was found that students used more than one type of home remedies at a time. Non-pharmacological methods are effective, easy to use, low cost and may be a valuable approach for dysmenorrhea management.^20^

The present study had some limitations. The findings of the current study was derived from single center with small sample size. The study included--only medical and paramedical undergraduate students. This also limits the possibility of generalizing the results. Being a student studying in medical and related discipline, and receiving training in various related subjects, such as Pharmacology, is likely to influence the treatment of dysmenorrhea and the ability to self-medicate. There is need of multicentric study with large sample of students from different disciplines, including both medical and other sciences, which would allow the results to be generalized and compared.

## CONCLUSIONS

The prevalence of self-medication in primary dysmenorrhea among undergraduate students was lower when compared to similar studies done in similar settings. Only few consult a healthcare professional and the majority of them choose to self-medicate to manage dysmenorrhea. The barriers to seek medical attention by dysmenorrheic students need to be explored. Effective measures in order to improve awareness towards self-medication practices should be implemented. Restriction of sale of drugs with potentially harmful effects should be implemented effectively with monitoring systems.
